# OliveNet™: a comprehensive library of compounds from *Olea europaea*

**DOI:** 10.1093/database/bay016

**Published:** 2018-02-12

**Authors:** Natalie P Bonvino, Julia Liang, Elizabeth D McCord, Elena Zafiris, Natalia Benetti, Nancy B Ray, Andrew Hung, Dimitrios Boskou, Tom C Karagiannis

**Affiliations:** 1 Epigenomic Medicine, Department of Diabetes, Central Clinical School, Monash University, Melbourne, VIC 3004, Australia; 2 Health Innovations Research Institute, School of Applied Sciences, RMIT University, VIC 3001, Australia; 3 McCord Research, Coralville, IA 52241, USA; 4 Aristotle University, Thessaloniki 54124, Greece; 5 Department of Pathology, The University of Melbourne, Parkville, VIC 3052, Australia

## Abstract

Accumulated epidemiological, clinical and experimental evidence has indicated the beneficial health effects of the Mediterranean diet, which is typified by the consumption of virgin olive oil (VOO) as a main source of dietary fat. At the cellular level, compounds derived from various olive (*Olea europaea*), matrices, have demonstrated potent antioxidant and anti-inflammatory effects, which are thought to account, at least in part, for their biological effects. Research efforts are expanding into the characterization of compounds derived from *Olea europaea*, however, the considerable diversity and complexity of the vast array of chemical compounds have made their precise identification and quantification challenging. As such, only a relatively small subset of olive-derived compounds has been explored for their biological activity and potential health effects to date. Although there is adequate information describing the identification or isolation of olive-derived compounds, these are not easily searchable, especially when attempting to acquire chemical or biological properties. Therefore, we have created the OliveNet™ database containing a comprehensive catalogue of compounds identified from matrices of the olive, including the fruit, leaf and VOO, as well as in the wastewater and pomace accrued during oil production. From a total of 752 compounds, chemical analysis was sufficient for 676 individual compounds, which have been included in the database. The database is curated and comprehensively referenced containing information for the 676 compounds, which are divided into 13 main classes and 47 subclasses. Importantly, with respect to current research trends, the database includes 222 olive phenolics, which are divided into 13 subclasses. To our knowledge, OliveNet™ is currently the only curated open access database with a comprehensive collection of compounds associated with *Olea europaea*.

Database URL: https://www.mccordresearch.com.au

## Introduction

The genus *Olea* (Oleaceae) contains approximately 40 taxa of evergreen shrubs and trees, found throughout southern Europe, Africa, Asia and Oceania (1). Native to the Mediterranean basin is *Olea europaea* is ubiquitously distributed throughout the region and is grown commercially for its fruit which contributes to the production of olive oil and table olives (2). Its hardiness and ubiquitous consumption have seen the cultivation of *O. europaea* endure from the Copper Age to the present day, where it is now one of the most valuable crops worldwide (3). Traditionally, the olive fruit and oil has been widely used in folk medicine, where it is used as a topical antiseptic and an analgesic for rheumatism and abdominal pain (4, 5). In more recent times, the beneficial health effects associated with the consumption of the extra-virgin olive has been highlighted by studies encompassing the Mediterranean diet, where olive oil is a primary source of dietary fat (6, 7).

**Figure 1.**
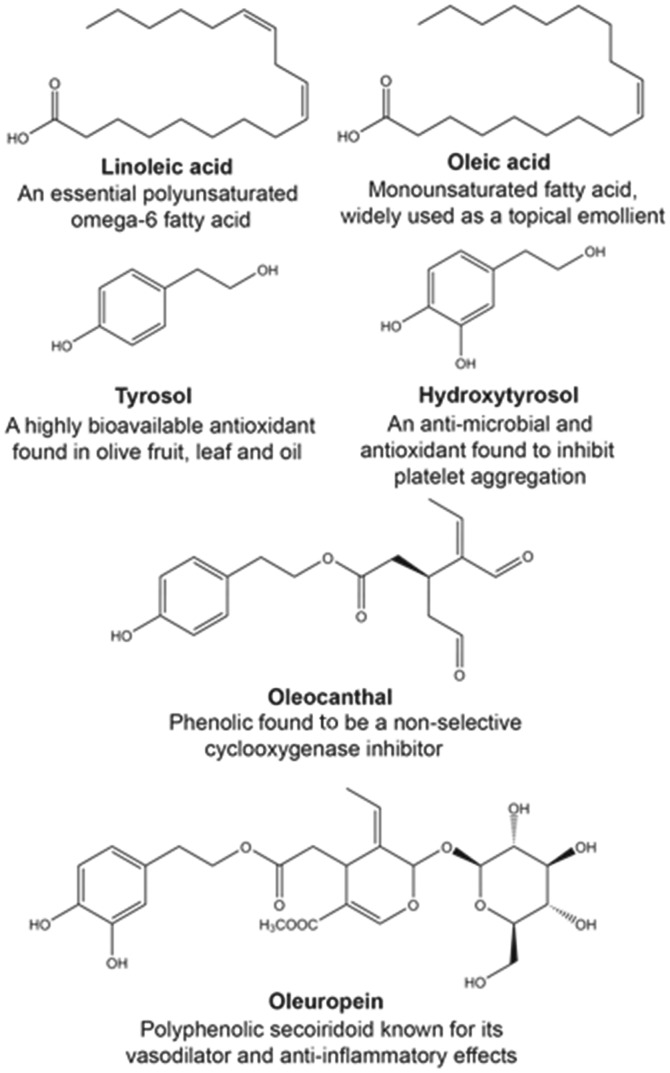
Chemical structures and key feature of widely investigated fatty acids (oleic and linoleic acids), and phenolic compounds (tyrosol, hydroxytyrosol, oleocanthal, and oleuropein) from *Olea europaea*.

Typified by the classical Seven Countries Study and more recently the Prevention with Mediterranean Diet study, the potential health benefits of the Mediterranean diet, associated with the consumption of extra-virgin olive oil (VOO), have emerged for a wide range of conditions including, cardiovascular disease, metabolic disease and diabetes, cancer prevention and neurodegeneration (8–14). In simple terms, extra-VOO is compromised of the major fatty acid fraction (98–99%), which comprised of predominantly oleic acid (55–83%) and linoleic acid (up to approximately 20%; Figure 1), and the minor constituents that incorporate the phenolic compounds (1–2%) (15).

Historically, the health benefits of extra-VOO was attributed to the high level of the mono- (oleic acid) and poly- (linoleic acid) unsaturated fatty acids, which, e.g. have been shown to have a wide range of beneficial cardiovascular effects (16–18). Further, it is now recognized that many of the beneficial effects of extra-VOO may be due to the unique minor compounds, including the flavonoids, lignans, secoiridoids and their hydrolysis products (19–21). These compounds have been shown to possess a broad range of bioactive properties, including antioxidant, anti-inflammatory and chemo-preventative activities, which have been elucidated over the past 20 years. The most widely investigated olive phenolic compounds include, oleuropein, hydroxytyrosol, tyrosol and oleocanthal (Figure 1). The potential beneficial health effects and cellular and molecular mechanisms of oleuropein, hydroxytyrosol and tyrosol have been reviewed extensively (22–29). Oleocanthal also represents an important phenolic compound in extra-VOO, which has been shown to possess potent antioxidant and anti-inflammatory effects, at least in part, through non-specific inhibition of cyclooxygenase-1 and -2 enzymes (30, 31).

Despite the promise of the Mediterranean diet with the consumption of extra-virgin olive and the encouraging results with specific fatty acids and phenolics, only a very small subset of compounds associated with *O. europaea* have been investigated to date. Importantly, many compounds that are structurally similar the well-known oleuropein, hydroxytyrosol, tyrosol and oleocanthal are either: (i) not commercially available, (ii) have not been synthesized in or isolated in quantities allowing meaningful experimentation and/or (iii) where available, have not been investigated for their biological effects.

## OliveNet™: construction and content

The considerable diversity and complexity of compounds derived from *O. europaea* as well as the matrix (leaf, fruit, oil, pomace and wastewater), in which they are found, has posed challenges for characterization and isolation of many of these compound. However, there is adequate information available in the literature describing the identification and isolation of compounds from the olive. Whilst found exclusively in the literature, these compounds are not easily searchable, especially when attempting to acquire specific information or finding compounds with specific chemical or biological properties. Further, it is difficult to decipher which compounds from the literature have been included in existing small-molecule databases, such as the most comprehensive, PubChem (32). Therefore, we created the comprehensive OliveNet™ database incorporating the individual compounds derived from *O. europaea*. Curated and fully referenced, the OliveNet™ database serves as a valuable resource for those within various scientific disciplines interested in compounds found within the various matrices of the olive, the associated analytical techniques used in their identification and/or isolation and, where appropriate key information related to known biological activities.

Overall, OliveNet™ describes 676 compounds associated with *O. europaea*, including 222 phenolic compounds. Compounds were identified from a comprehensive review of 181 scientific publications, including journal articles and books, using relevant search terms including ‘olea europaea’, ‘olive’, ‘phenol’, ‘polyphenol’ in PubMed and Science Direct. Overall, the data entry goal was to source, extract and critically assess data from published reports concerned with the identification and biological effects of bioactive compounds in *O. europaea.* Original publications were selected based on the analytical methods used to identify and quantify the compounds present in the olive matrices. These involved a range of extraction processes, analytical separation and quantification techniques. Generally, high-performance liquid chromatography coupled to mass spectrometry/gas chromatography was employed to separate and then quantify the unsaponifiable compounds, including the phenolics (33–35). High resolution multinuclear (^1 ^H, ^13 ^C, ^31 ^P) nuclear magnetic resonance spectroscopy was also used for elucidation of isolated compounds (36–39). These techniques represent a higher sensitivity compared to other spectrophotometric techniques, which have several limitations associated with their application (40). The importance of appropriate methodologies for isolation and characterization of bioactive phytonutrients such as phenolic compounds and flavonoids, which are included in our database, have been thoroughly reviewed, and research efforts for improving the quality of extracts are constantly evolving (41–45). For our OliveNet™ database, if the methodology was not sufficiently documented or considered inadequate, the compound was excluded, resulting in the reduction of the total 752 compounds initially identified to the final 676 individual compounds catalogued in the database.

**Figure 2.**
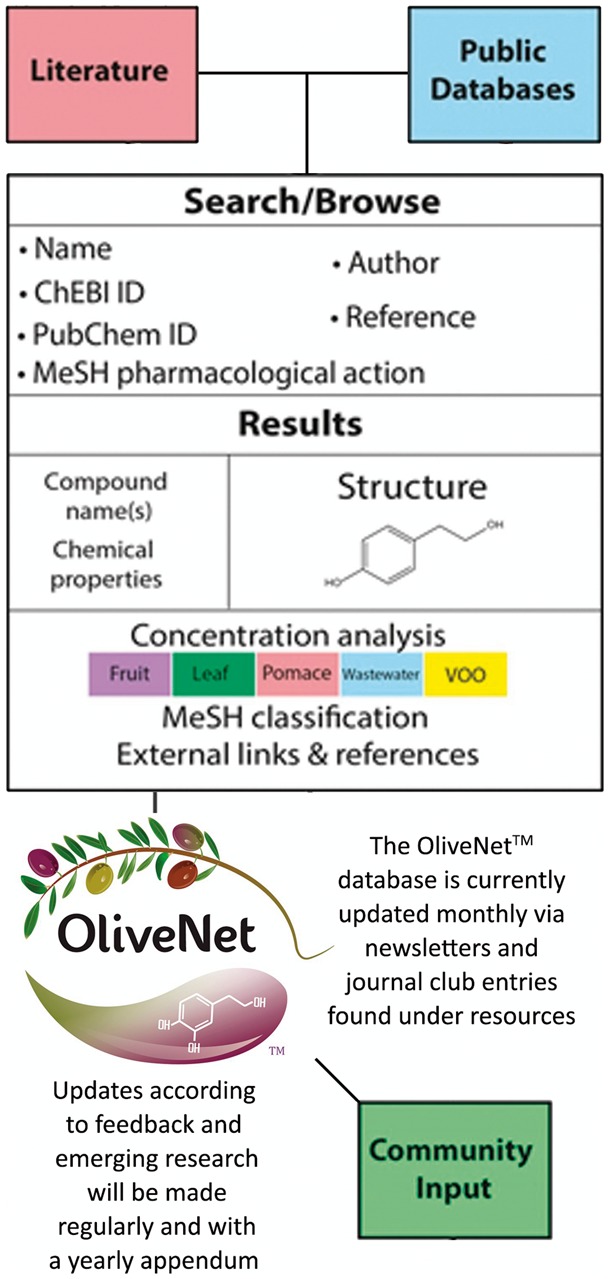
Integration of structure and composition of the OliveNet™ database. Compounds were identified from a comprehensive review of 181 scientific publications and compound information was obtained from public databases (PubChem, HMDB and ChEBI). Together with the chemical structure and basic chemical analyses, MeSH terms are provided where available. Concentrations of each compound in fruit, leaf, pomace, wastewater, VOO are indicated where available. Known biological effects and pharmacological activity of the compounds are included with references. As further relevant research emerges, we can easily update compounds and biological activities via community input.

Compound information regarding pharmacological activity was obtained from PubChem, Human Metabolome Database (HMDB) and Chemical Entities of Biological Interest (ChEBI), with links provided (46–48). Further, medical subject headings (MeSH) terms are provided where available. Similarly, concentrations of each compound in the various olive matrices (leaf, fruit, oil, pomace and wastewater) are documented where available. Known biological effects and pharmacological activity of the compounds are included, with references to biological efficacy determined by *in vitro* or *in vivo* studies as required. Important features of the OliveNet™ database are that it (i) easily adapted to include new compounds and (ii) encourages community input (Figure 2). Therefore, as further relevant research emerges, we can easily update compounds and biological activities.

## Composition of OliveNet™

**Figure 3.**
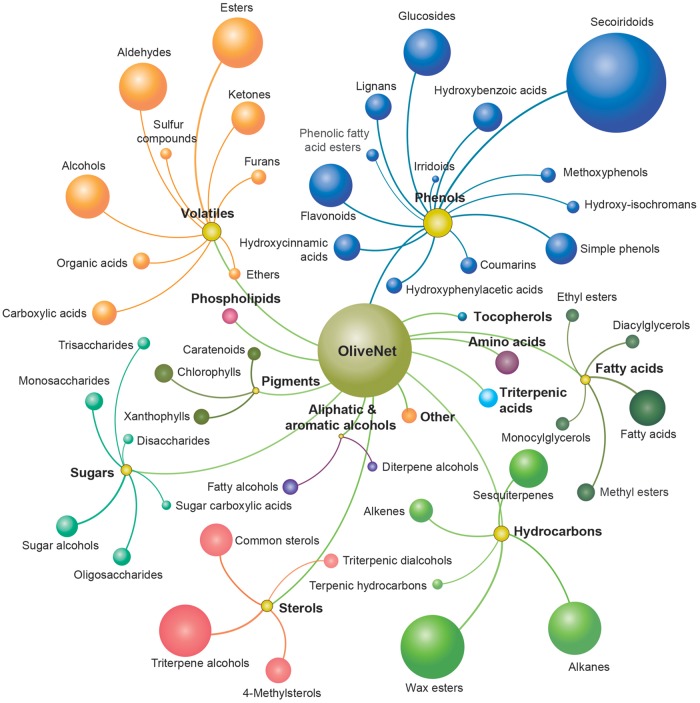
The OliveNet™ database consists of a total of 676 individual compounds categorized into 13 classes (bold and colour coordinated) and 47 subclasses (size of ball reflects the number of compounds in each subclass).

The 676 distinct chemical compounds derived from *O. europaea* in the OliveNet™ database are divided into 13 main classes, namely phenols, fatty acids, aliphatic and aromatic alcohols, sterols, phospholipids, triterpenic acids, volatiles, hydrocarbons, sugars, pigments, tocopherols, amino acids and a small number of other unclassified compounds (Figure 3). As described, 181 individual references were utilized to identify and classify the chemical compounds. The detailed classification process and the pertinent references for each compound have been documented in a table entitled ‘Original OliveNet™ Library: Quick Reference’, which can be easily be obtained from the web site. The table contains the complete list of compounds in their rightful overall class, an indication of the olive matrix in which they have been identified, and the key reference associated with the classification process.

The phenolic class of compounds is of particular importance with respect to human nutrition. Compounds in this class contribute to the stability and organoleptic properties of the oil and possess a range of biological activities, which are of particular importance. Indeed, the European Food Safety Authority has approved claims stating that the dietary intake of extra-VOO phenolic compounds prevent oxidation of low-density lipoprotein, display anti-inflammatory properties and contribute to body defences against external agents (49). As described earlier, research has focussed on a few phenolic compounds such as hydroxytyrosol and oleuropein, which have been shown to prevent oxidative stress and selectively induce cell-cycle arrest and induce apoptosis in cancer cells (50–54). It is generally recognized that their orthodiphenolic structure confers strong antioxidant activity, while their lipophilicity profiles provide insight into their anti-inflammatory function (51, 55).

**Figure 4.**
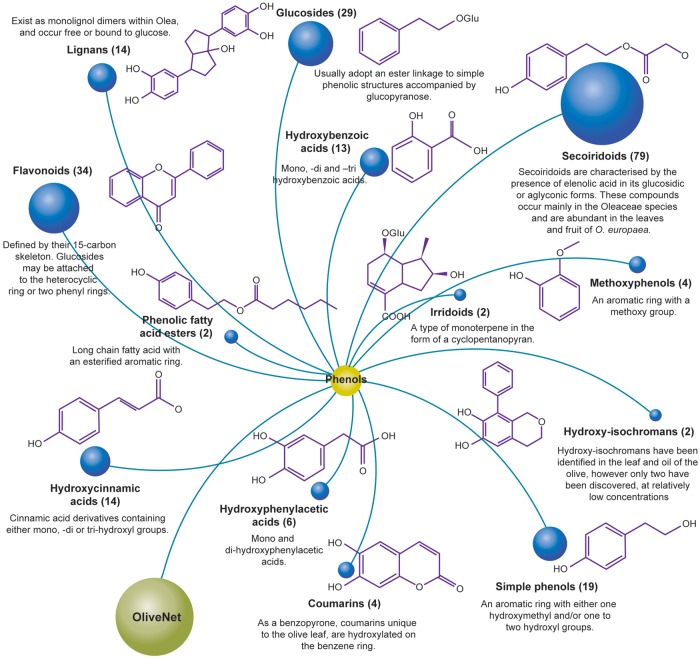
The OliveNet™ database consists of 222 individual phenolic compounds divided into 13 subclasses. The number of individual compounds in each subclass, representative chemical structures, and relevant key information are shown.

Phenolic compounds encompass a diverse subset of chemical structures found within the leaf, fruit, oil, pomace and wastewater from processing. Structurally, they are characterized by an aromatic ring with one or more hydroxyl groups. In the OliveNet™ database, we describe a total of 222 phenolic compounds derived from *O. europaea*, which are further divided into 13 subclasses comprised of the: simple phenols, methoxyphenols, hydroxybenzoic acids, hydroxyphenylacetic acids, hydroxycinnamic acids, secoiridoids, glucosides, flavonoids, hydroxyisochromans, coumarins, irridoids, lignans and phenolic fatty acid esters (Figure 4). Among the phenolic compounds presented in OliveNet™, approximately 45% are not currently commercially available and only a small subset has been investigated in biological experiments. For example, one of the most widely investigated olive phenolics is hydroxytyrosol and as can identified in the ‘Original OliveNet™ Library: Quick Reference’ table on the website, hydroxytyrosol, is a simple phenolic compound found in various concentrations in the olive fruit, leaf, pomace, and in extra-VOO (56–59). In contrast, much less is known about other more obscure olive-derived phenolics, such as dihydro-p-coumaric acid (phloretic acid), certain hydroxyphenylacetic acids (e.g. 4-hydroxy-3-methoxyphenylacetic acid, and caffeoylglucose, to name a few examples (60–62). In this context, one of the major aims of producing the OliveNet™ database is to identify and classify compounds to inspire potential synthesis of non-commercially available compounds and to encourage further exploration of the less well-studied compounds.

### Website content and utility

**Figure 5.**
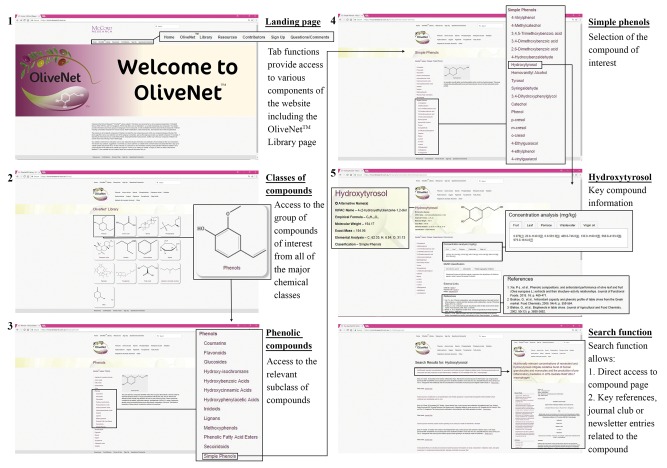
Screenshots from the OliveNet™ database. An example of navigation from the OliveNet™ Library homepage, to the phenolic compound landing page, and ultimately to an individual phenolic compound page (in this example, hydroxytyrosol), is shown. The direct search function is also highlighted

OliveNet™ employs a web interface, which is simple and easy to use and allows for compounds to be searched by a number of different criteria. As the step-by-step workflow indicates users may browse for compounds of interest via the OliveNet™ Library homepage (Figure 5). Alternatively, direct website search engine is provided on each page, where compound names or keywords can be entered directly (Figure 5). Users are initially directed to the site homepage, where user-friendly links for searching and browsing compounds are provided. On each compound information page, the chemical structure is displayed and all of the chemical names, including the IUPAC name, common name and any synonyms for that particular compound are listed. The compound properties, including the molecular weight, exact mass and elemental analysis is also provided. Where available, the concentration (mg/kg) quantified in the leaf, fruit, oil and in the oil processing products (pomace and wastewater), is provided with relevant references. Similarly, where relevant, references are provided in relation to the classification and bioactivity of individual compounds.

Overall, the OliveNet™ database provides a comprehensive record of compounds associated with *O. europaea* in one easily searchable database. We intend OliveNet™ to be a valuable resource for those conducting chemical, biochemical or biological research related to *O. europaea*. It is anticipated that primary users will be from the food industry where structural analyses, synthesis of novel compounds and chemical characterization, may ultimately lead to additional standards for assessment of the quality of extra-VOOs. More broadly in the biological field, OliveNet™ is intended to serve as a platform for initial structure-activity relationship analyses, *in silico* modeling and characterization to preferred biological pathway targets and potentially identification of lead compounds for isolation or synthesis. Ultimately, novel compounds may emerge for various diseases and these potentially suitable candidates may be isolated or synthesized in experimentally meaningful purities and concentrations, for further *in vitro* and *in vivo* exploration and validation.

With respect to community input and evolution of the website, with further research and understanding, additional compounds, or even classes of compounds will incorporated into the database. For example, we are currently scoping the seleno-amino acids and phytoprostanes, which, represent classes of important bioactive compounds (63–65). The antioxidant and anti-inflammatory properties of seleno-amino acids (such as selenocysteine) have been widely studied and their presence in extra-VOO is emerging (66, 67). Similarly, phytoprostanes are emerging as important bioactive compounds found in extra-VOO and these also targets for inclusion in the database (68–70).

### Conclusions and future enhancements

In summary, OliveNet™ is a freely available database containing compounds identified from *O. europaea*. As an online interface, OliveNet™ allows olive researchers and industry users to easily find information about 676 common and mostly obscure compounds associated with *O. europaea*, as well as to examine their chemical structure for structure-activity relationship analyses and for potential use in virtual screening. OliveNet™ represents a comprehensively referenced database linked to peer-reviewed literature, which will be updated as required. Further, modifications and additions to reflect new discoveries from the research community are encouraged. It is anticipated that as analytical techniques advance, the integration of past and future work by various disciplines will drive forward the validation of possible bioactives. Since there are still many unknown compounds present in the polar fraction of olive oil, it remains important to carry out fundamental chemical analyses of the various *O. europaea* matrices. New findings can be easily incorporated into the OliveNet™ database.
